# Grip Strength in Patients with Gastrointestinal Diseases

**DOI:** 10.3390/jcm11082079

**Published:** 2022-04-07

**Authors:** Ken Asaishi, Masahiro Matsui, Hiroki Nishikawa, Masahiro Goto, Akira Asai, Kosuke Ushiro, Takeshi Ogura, Toshihisa Takeuchi, Shiro Nakamura, Kazuki Kakimoto, Takako Miyazaki, Shinya Fukunishi, Hideko Ohama, Keisuke Yokohama, Hidetaka Yasuoka, Kazuhide Higuchi

**Affiliations:** 1 The Second Department of Internal Medicine, Osaka Medical and Pharmaceutical University, Takatsuki 569-8686, Japan; ken.asaishi@ompu.ac.jp (K.A.); masa1987_11_18@yahoo.co.jp (M.M.); masahiro.goto@ompu.ac.jp (M.G.); akira.asai@ompu.ac.jp (A.A.); ushiro.1989@icloud.com (K.U.); oguratakeshi0411@yahoo.co.jp (T.O.); toshihisa.takeuchi@ompu.ac.jp (T.T.); shiro.nakamura@ompu.ac.jp (S.N.); kazuki.kakimoto@ompu.ac.jp (K.K.); takako.miyazaki@ompu.ac.jp (T.M.); shinya.fukunishi@ompu.ac.jp (S.F.); hideko.ohama@ompu.ac.jp (H.O.); hammer_0906@yahoo.co.jp (K.Y.); yh0403.4351@gmail.com (H.Y.); kazuhide.higuchi@ompu.ac.jp (K.H.); 2 The Premier Departmental Research of Medicine, Osaka Medical and Pharmaceutical University, Takatsuki 569-8686, Japan

**Keywords:** grip strength, gastrointestinal disease, aging, malnutrition, inflammation

## Abstract

We sought to elucidate factors contributing to the grip strength (GS) decline in patients with gastrointestinal diseases (Ga-Ds, *n* = 602, 379 males, median age = 72 years). The GS decline in males and females was defined as <28 kg and <18 kg, respectively, following the current Asian guidelines. The median GS (male) was 28.8 kg, and GS decline (male) was found in 169 patients (44.6%). The median GS (female) was 17.5 kg, and GS decline (female) was found in 122 patients (54.7%). Advanced cancer was identified in 145 patients (24.1%). In terms of the univariate analysis of parameters of the GS decline, age (*p* < 0.0001), gender (*p* = 0.0181), body mass index (BMI, *p* = 0.0002), ECOG-PS (*p* < 0.0001), SARC-F score (*p* < 0.0001), hemoglobin value (*p* < 0.0001), total lymphocyte count (*p* < 0.0001), serum albumin value (*p* < 0.0001), C reactive protein (CRP) value (*p* < 0.0001), and estimated glomerular filtration rate were statistically significant. In terms of the multivariate analysis, age (*p* < 0.0001), BMI (*p* = 0.0223), hemoglobin value (*p* = 0.0186), serum albumin value (*p* = 0.0284), the SARC-F score (*p* = 0.0003), and CRP value (*p* < 0.0001) were independent parameters. In conclusion, the GS decline in patients with Ga-Ds is closely associated with not only the primary factor (i.e., aging) but also secondary factors such as inflammatory factors and nutritional factors.

## 1. Introduction

Skeletal muscle, besides controlling body movement, functions as a store of protein and has an essential role in glucose uptake and energy metabolism [1,2]. Sarcopenia is characterized by progressive skeletal muscle mass loss and decline in function, linking to physical frailty and survival [1,2,3]. Malnutritional status, inactivity, advanced cancer burden, and persistent inflammatory state often seen in gastrointestinal diseases (Ga-Ds) are common features for sarcopenia [1,2,3,4,5,6,7,8,9,10]. Loss of dietary intakes and worsening of nutritional condition can be also frequently encountered in patients with Ga-Ds [9], and sarcopenia in patients with Ga-Ds is associated with decreased quality of life (QOL) and adverse outcomes [9,11]. Ga-D is a typical disease that causes secondary sarcopenia owing to the disease burden itself [11].

In recent years, a lot of studies have presented that grip strength (GS) is related to the onset of disease and life expectancy [12,13,14,15]. The results of a study in the UK that examined the relationship between GS, disease onset, and mortality were reported [16]. The study included 502,293 people aged 40–69 years, classified into four categories based on the baseline GS, and examined the relationship between GS and overall survival, cardiovascular disease-related survival, respiratory disease-related survival, and cancer-related survival [16]. The results showed that for every 5 kg decrease in GS, overall mortality increased by 20%, cardiovascular disease mortality increased by 19%, respiratory disease mortality increased by 31%, cancer mortality increased by 17%, and this relationship was particularly strong among young people [16]. In addition, the incidence and mortality rates of cardiovascular diseases, respiratory diseases, and cancers were all higher in people with GS of less than 26 kg in men and less than 16 kg in women. In other words, lower GS was as likely an indicator of future disease development and mortality as higher blood pressure and obesity, which are well-accepted prognostic markers [16].

The progression of muscle strength decline is usually accompanied by various metabolic changes [8]. As far as we are aware, however, there have been few reports with regard to factors contributing to the GS decline in patients with Ga-Ds. These problems may deserve to be solved, which urged us to conduct the current cross-sectional analysis.

## 2. Patients and Methods

### 2.1. Patients

In our hospital, between October 2020 and October 2021, 602 Japanese Ga-D hospitalized patients with data for GS were identified. In our hospital, GS has been routinely tested in each patient on admission, and sarcopenia risk was assessed according to the SARC-F. SARC-F is recommended for active use as an initial screening method for sarcopenia in the current globally accepted guidelines [17,18]. SARC-F is taken from S for Strength, A for Assistance walking, R for Rising from chair, C for Climbing stairs, and F for Falls, and each is rated on a scale of 0 to 2, with the total score being calculated [19,20,21,22]. In the Asian guidelines for sarcopenia, the recommended reference value of the SARC-F score for the presence of sarcopenia is 4 [17]. As a rule, each inpatient was required to be included in the SARC-F questionnaire on admission. GS was tested according to the guidelines [8]. In brief, the average of the left and right GS was used. The GS decline in male and female was defined as <28 kg and <18 kg, respectively, following the international guidelines [17].

### 2.2. Study Procedure and Ethics

First, the relevance between the GS decline and baseline characteristics was investigated. Next, univariate and multivariate analyses of the GS decline were carried out. Finally, receiver operating characteristic curve (ROC) analysis of the GS decline was enforced. Advanced cancer was defined as stage III or far advanced cancer. The study was retrospective in design, approved by the relevant institutional ethics committee (approval number, 2021-122), and conducted in accordance with the Declaration of Helsinki. As this study was a retrospective observational study, patients’ consent was waived.

### 2.3. Statistics

For the comparison between two groups of continuous variables, the most appropriate choice between the Student’s *t* test and the Mann–Whitney *U* test was selected after confirmation of distribution. Continuous variables were presented as median (interquartile range, IQR) unless otherwise mentioned. In between-group comparisons of nominal variables, the Pearson χ^2^ test was utilized. In the univariate analysis, effect size was also presented. Multivariate logistic regression analysis for the GS decline was also conducted to choose independent parameters considering significant parameters in the univariate analysis. ROC analysis for calculating the area under the ROC (AUC) was also done, and the reference value was set up as the point at which the sum of sensitivity and specificity is maximized. A *p* value less than 0.05 was the significant level in this study according to the JMP ver. 15 (SAS Institute Inc., Cary, NC, USA).

## 3. Results

### 3.1. Patient Baseline Features

Table 1 presents baseline features of all study subjects (*n* = 602, 379 males and 223 females, median (IQR) age = 72 (64–79) years). The median (IQR) GS in males was 28.8 (23.6–34.3) kg, and GS decline in males (i.e., <28 kg) was found in 169 patients (44.6%). The median (IQR) GS in females was 17.5 (13.9–20.5) kg, and GS decline in females (i.e., <18 kg) was found in 122 patients (54.7%). The median (IQR) body mass index (BMI) was 21.9 (19.6–24.4) kg/m^2^ (missing data, *n* = 2). The median (IQR) ECOG-PS was 0 (0–1). EOCG-PS 0 was found in 399 patients (66.3%), 1 in 108 (17.9%), 2 in 40 (6.6%), 3 in 38 (6.3%) and 4 in 17 (2.8%). The median (IQR) SARC-F score was 0 (0–2). There were 345 cases (57.3%) with a SARC-F score of 0, 99 (16.4%) with a score of 1, 52 (8.6%) with a score of 2, 33 (5.5%) with a score of 3, and 73 (12.1%) with a score of ≥4. Upper gastrointestinal disease (U-GD) was seen in 149 patients (advanced cancer, 37 cases (24.8%)), lower gastrointestinal disease (L-GD) in 156 (advanced cancer, 28 cases (18.0%)), biliary and pancreatic disease (BP-D) in 209 (advanced cancer, 54 cases (25.8%)), and liver disease (L-D) in 88 (advanced cancer, 26 cases (29.6%)). Overall, advanced cancer was identified in 145 patients (24.1%). Other than advanced cancer, diseases seen in patients included early-stage cancer and benign diseases such as chronic inflammatory disease, benign tumor, and infectious disease.

### 3.2. GS Decline and ECOG-PS and BMI

**Figure 1:**
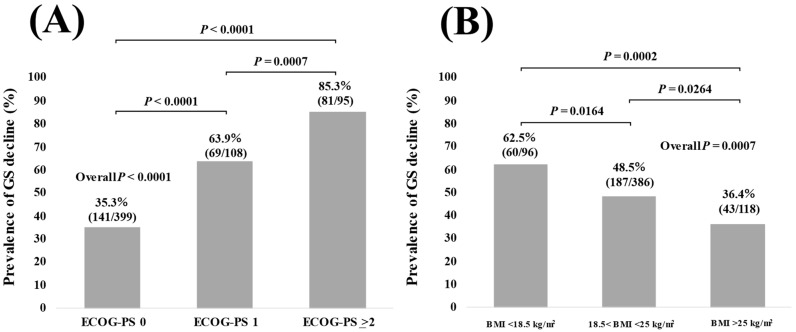
(**A**) The percentage of GS decline in patients with ECOG-PS 0 (*n* = 399), ECOG-PS 1 (*n* = 108), and ECOG-PS 2 (*n* = 95). (**B**) The percentage of GS decline in patients with BMI (kg/m^2^) < 18.5 (*n* = 96), 18.5 < BMI (kg/m^2^) < 25 (*n* = 386), and BMI (kg/m^2^) > 25 (*n* = 118).

In patients with ECOG-PS 0 (*n* = 399), 1 (*n* = 108), and ≥2 (*n* = 95), the percentage of GS decline was 35.3% (141/399), 63.9% (69/108), 85.3% (81/95) (*p* < 0.0001 in ECOG-PS 0 vs. 1, *p* < 0.0001 in ECOG-PS 0 vs. ≥2, *p =* 0.0007 in ECOG-PS 1 vs. ≥2, and overall *p* < 0.0001) (Figure 1A).

BMI (kg/m^2^) was classified into three groups (BMI (kg/m^2^) < 18.5, 18.5 < BMI (kg/m^2^) < 25, and BMI (kg/m^2^) > 25) based on the current guidelines in the Japanese Society for the Study of Obesity (JASSO) [23]. In patients with BMI (kg/m^2^) < 18.5 (*n* = 96), 18.5 < BMI (kg/m^2^) < 25 (*n* = 386), BMI (kg/m^2^) > 25 (*n* = 118), the percentage of GS decline was 62.5% (60/96), 48.5% (187/386), 36.4% (43/118) (*p* = 0.0164 in BMI (kg/m^2^) < 18.5 vs. 18.5 < BMI (kg/m^2^) < 25, *p* = 0.0264 in 18.5 < BMI (kg/m^2^) < 25 vs. BMI (kg/m^2^) > 25, *p* = 0.0002 in BMI (kg/m^2^) < 18.5 vs. BMI (kg/m^2^) > 25, and overall *p* = 0.0007) (Figure 1B).

### 3.3. GS Decline and the SARC-F Score

**Figure 2:**
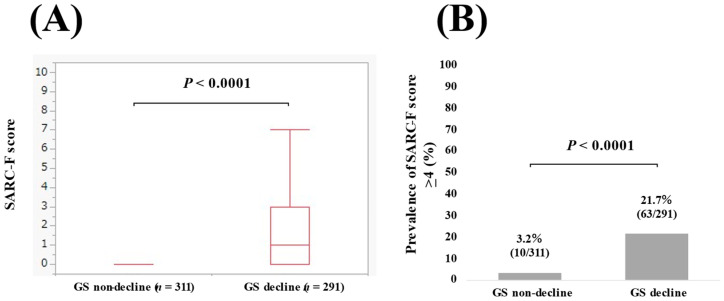
(**A**) The SARC-F score in patients with the GS non-decline (*n* = 311) and the GS decline (*n* = 291). (**B**) The percentage of the SARC-F score ≥ 4 in patients with the GS non-decline and the GS decline.

The median (IQR) SARC-F score in patients with GS decline (*n* = 291) and without GS decline (*n* = 311) was 1 (0–3) and 0 (0–0) (*p* < 0.0001, Figure 2A). The percentage of patients with a SARC-F score ≥ 4 in patients with and without GS decline was 21.7% (63/291) and 3.2% (10/311) (*p* < 0.0001, Figure 2B).

### 3.4. GS Decline According to the Primary Origin of the Disease

The percentage of GS decline in patients with U-GD, L-GD, BP-D, and L-D was 53.7% (80/149) in U-GD, 39.7% (62/156) in L-GD, 49.3% (103/209) in BP-D, and 52.3% (46/88) in L-D (overall *p* = 0.0737). 

### 3.5. GS Decline and the SARC-F Score in Patients with and without Advanced Cancer

**Figure 3:**
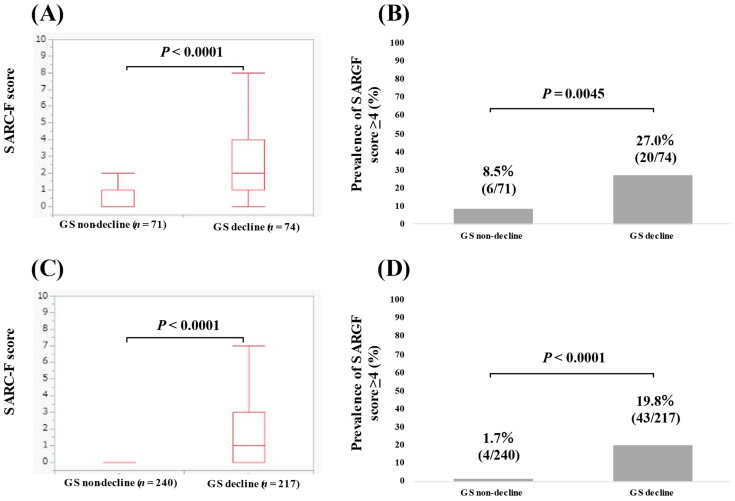
(**A**) The SARC-F score in patients with GS non-decline (*n* = 71) and GS decline (*n* = 74) (advanced cancer cases, *n* = 145). (**B**) The percentage of the SARC-F score ≥ 4 in patients with GS non-decline and GS decline (advanced cancer cases). (**C**) The SARC-F score in patients with GS non-decline (*n* = 240) and GS decline (*n* = 217) (non-advanced cancer cases, *n* = 457). (**D**) The percentage of the SARC-F score ≥ 4 in patients with GS non-decline and GS decline (non-advanced cancer cases).

In patients with advanced cancer (*n* = 145), the median (IQR) SARC-F score in patients with GS decline (*n* = 74) and without GS decline (*n* = 71) was 2 (1–4) and 0 (0–1) (*p* < 0.0001, Figure 3A). The percentage of patients with a SARC-F score ≥ 4 in patients with and without GS decline was 27.0% (20/74) and 8.5% (6/71) (*p* = 0.0045, Figure 3B).

In patients without advanced cancer (*n* = 457), the median (IQR) SARC-F score in patients with GS decline (*n* = 217) and without GS decline (*n* = 240) was 1 (0–3) and 0 (0–0) (*p* < 0.0001, Figure 3C). The percentage of patients with a SARC-F score ≥ 4 in patients with and without GS decline was 19.8% (43/217) and 1.7% (4/240) (*p* < 0.0001, Figure 3D).

### 3.6. GS Decline According to the Primary Disease Site in Patients with and without Advanced Cancer

Among four categories of primary origin in patients with advanced cancer, no significant difference was seen in the percentage of GS decline (62.2% (23/37) in U-GD, 32.1% (9/28) in L-GD, 53.7% (29/54) in BP-D, 50.0% (13/26) in L-D, overall *p* = 0.1117).

Among four categories of primary origin in patients without advanced cancer, no significant difference was seen in the percentage of GS decline (50.9% (57/112) in U-GD, 41.4% (53/128) in L-GD, 47.7% (74/155) in BP-D, 53.2% (33/62) in L-D, overall *p* = 0.3558).

### 3.7. Uni- and Multivariate Analysis of Variables for the GS Decline

In the univariate analysis of variables for the GS decline, age (*p* < 0.0001), gender (*p* = 0.0181), BMI (*p* = 0.0002), ECOG-PS (*p* < 0.0001), SARC-F score (*p* < 0.0001), hemoglobin value (*p* < 0.0001), total lymphocyte count (*p* < 0.0001), serum albumin value (*p* < 0.0001), C reactive protein (CRP) value (*p* < 0.0001), and estimated glomerular filtration rate were statistically significant (Table 2). In the multivariate analysis, age (*p* < 0.0001), BMI (*p* = 0.0223), hemoglobin value (*p* = 0.0186), serum albumin value (*p* = 0.0284), the SARC-F score (*p* = 0.0003), and CRP value (*p* < 0.0001) were independent parameters of the GS decline (Table 3). Table 3 involves hazard ratio and 95% confidence interval in each factor.

### 3.8. ROC Analysis of Independent Parameters for the GS Decline

ROC analysis of independent factors in the multivariate analysis for GS decline was performed. AUC, the sensitivity, the specificity, and optimal reference value in each factor are demonstrated in Table 4. Age had the highest AUC for the GS decline (AUC = 0.74), followed by the SARC-F score (AUC = 0.73). The optimal reference value of the SARC-F score was 1, but when the reference value of the SARC-F score was changed to 4 (recommended reference value in the Asian guidelines), the sensitivity and specificity was 21.7% and 96.8%.

## 4. Discussion

Skeletal muscle mass and strength decrease from middle age to old age. Research related to sarcopenia has been progressing rapidly around the world in recent years. New evidence is also emerging on drug interventions for sarcopenia [24,25]. While in the COVID-19 pandemic, muscle strength decline is a serious concern due to decreased physical activity in daily life [26]. Many studies have shown that GS is related to the overall strength of the whole body, and GS decline is associated with decreased physical activity and adverse prognosis [12,13,14,15,27]. In our previous study, decreased GS was an independent adverse predictor for overall survival in patients with chronic liver disease (*n* = 1624) [27]. However, to our knowledge, few reports have addressed the contributing factors for GS decline in patients with Ga-Ds. To clarify these issues appears to be clinically meaningful. A large cohort (*n* = 602) is a major strength in the current analysis.

In our results, older age, lower BMI, lower hemoglobin, lower serum albumin, higher SARC-F score, and higher CRP level were independent factors for the GS decline in our multivariate analysis. These results imply that the GS decline in Ga-Ds is closely associated with not only the primary factor (i.e., aging) but also secondary factors such as inflammatory factor and nutritional factor. SARC-F involves strength (S, “How difficult is it to lift or carry an object weighing around 4.5 kg?”) [19,20], and thus it is not so surprising that the SARC-F score was an independent parameter of the GS decline. Advanced cancer and primary site of disease were not independent factors for the GS decline. The location of the primary lesion and the degree of disease progression do not seem to be directly related to the GS decline.

In our data, the median GS in males and females was 28.8 kg and 17.5 kg, which was considerably lower than that in Japanese people aged >60 years (average GS: male, 38.8 kg; female, 24.2 kg [28]). Multiple factors related to the GS decline in Ga-Ds may account for the current results. On the other hand, out of patients with the GS decline (*n* = 291), the average SARC-F score in patients with BMI (kg/m^2^) < 18.5 (*n* = 60), 18.5 < BMI (kg/m^2^) < 25 (*n* = 187), and BMI (kg/m^2^) > 25 (*n* = 43) was 1.7, 1.9 and 2.1, respectively. These results indicated that patients with the GS decline and BMI > 25 kg/m^2^ had the highest possibility for the definite diagnosis of sarcopenia. Sarcopenic obesity may be a serious concern in clinical settings because of its prognostic impact [29,30,31]. For Japanese people, a BMI of 22 kg/m^2^ is said to be the least likely to cause illness [32]. In our ROC analysis of BMI for the GS decline, the optimal reference value of BMI was 22.6 kg/m^2^, which is almost identical to the previous reports [32].

CRP value was an independent predictor for the GS decline. A recent meta-analysis reported that independent of disease state, higher CRP values were associated with GS decrease and knee extension strength decrease, which is in line with our data [33]. Proinflammatory cytokines such as TNF-α, IL-1β, IL-6, IFN-γ, and chemokines can cause increment in energy expenditure, appetite loss, and reduced dietary intake, which can be also linked to the current results [27]. Appropriate nutritional interventions may be considered for patients with higher CRP values [34]. Cachexia is common in patients with diseases related to chronic inflammation and skeletal muscle mass loss [35]. Although cachexia is common in patients with Ga-Ds, it is not well recognized by medical experts, and in most cases, the nutritional status deteriorates further without being diagnosed, resulting in skeletal muscle mass loss and decreased physical function [36,37]. In terms of SARC-F, the optimal reference value of the SARC-F score was 1 (the sensitivity/specificity, 64.3/77.5%), but when the reference value of the SARC-F score was changed to 4 (recommended reference value in the Asian guidelines [17]), the sensitivity/specificity was 21.7/96.8%. The relatively low sensitivity of the SARC-F score to the diagnosis of sarcopenia is a concern, which is identical to the current results [38,39,40]. The recommended reference value of the SARC-F score may be re-considered. On the other hand, lower hemoglobin value was independently linked to the GS decline in our data. A Korean study demonstrated a strong association between lower GS and anemia, which is in line with our data [41].

It should be understood that there are several limitations to the present analysis. First, this study is a single-center, cross-sectional study and is retrospective in nature. Second, because data for skeletal muscle mass was not available in the current analysis, the exact number of subjects with a confirmed diagnosis for sarcopenia was unknown. Third, the cohort in this study was highly heterogeneous, including a wide range of Ga-Ds (i.e., U-GD, L-GD, BP-D, and L-D). Fourth, patient daily activities or dietary habits, which can affect GS, were not analyzed in this study, also creating bias. Fifth, effect size was relatively small throughout analyzed parameters. Nevertheless, our study results denoted that GS can be influenced by various factors other than aging in patients with GDs. In conclusion, we would like to emphasize this as an important point when considering the GS decline in patients with Ga-Ds.

## Figures and Tables

**Table 1:** Baseline characteristics (*n* = 602).

Variables	Number or Median (IQR)
Age (years)	72 (64–79)
Gender, male/female	379/223
ECOG-PS	0 (0–1)
Grip strength (male, kg)	28.8 (23.6–34.3)
Grip strength (female, kg)	17.5 (13.9–20.5)
The SARC-F score	0 (0–2)
Body mass index (kg/m^2^)	21.9 (19.6–24.4)
Advanced cancer, yes/no	145/457
Hemoglobin (g/dL)	12.5 (11.1–13.8)
Platelet count (×10^4^/μL)	21.9 (16.6–27.7)
Total lymphocyte count (/μL)	1330 (989–1822)
ALT (IU/L)	19 (13–32)
eGFR (mL/min/1.73 m^2^)	67 (55–81)
Serum albumin (g/dL)	3.8 (3.4–4.2)
C reactive protein (mg/dL)	0.18 (0.05–0.95)

IQR, interquartile range; ALT, alanine aminotransferase; eGFR, estimated glomerular filtration rate.

**Table 2:** Univariate analysis of factors associated with the grip strength decline.

Variables	Grip Strength Decline (*n* = 291)	Grip Strength Non-Decline (*n* = 311)	*p* Value	Effect Size
Age (years)	77 (71–82)	68 (56–74)	<0.0001	0.15
Gender (male/female)	169/122	210/101	0.0181	0.10
BMI (kg/m^2^)	21.1 (19.0–23.9)	22.7 (20.1–24.9)	0.0002	0.15
ECOG-PS	1 (0–2)	0 (0–0)	<0.0001	0.36
Advanced cancer, yes/no	74/217	71/240	0.5045	0.03
Primary origin of disease U-GD/L-GD/BP-D/L-D	80/62/103/46	69/94/106/42	0.0737	0.11
SARC-F score	1 (0–3)	0 (0–0)	<0.0001	0.38
Hemoglobin (g/dL)	11.7 (10.6–13.1)	13.2 (11.8–14.3)	<0.0001	0.33
Platelet count (×10^4^/μL)	21.4 (15.8–28.0)	22.4 (17.6–27.5)	0.4667	0.0
Total lymphocyte count (/μL)	1251 (900–1728)	1480 (1092–1890)	<0.0001	0.16
Serum albumin (g/dL)	3.6 (3.1–4.0)	4.0 (3.7–4.3)	<0.0001	0.32
CRP (mg/dL)	0.30 (0.07–2.32)	0.13 (0.05–0.41)	<0.0001	0.19
ALT (IU/L)	19 (12–32)	19 (13–32)	0.7145	0.01
eGFR (mL/min/1.73 m^2^)	63 (51–79)	71 (58–82)	0.0003	0.15

Data are expressed as number or median value (interquartile range). BMI, body mass index; U-GD, upper gastrointestinal disease; L-GD, lower gastrointestinal disease; BP-D, biliary pancreatic disease; L-D, liver disease; CRP, C reactive protein; ALT, alanine aminotransferase; eGFR, estimated glomerular filtration rate.

**Table 3:** Multivariate analysis of factors for the grip strength decline.

Variables	Multivariate Analysis		
	HR	95% CI	*p* Value
Age (per one year)	1.052	1.033–1.072	<0.0001
BMI (per one kg/m^2^)	0.938	0.888–0.992	0.0223
ECOG-PS (per one)	1.221	0.857–1.740	0.2678
Gender (female)	1.294	0.852–1.966	0.2268
SARC-F score (per one)	1.467	1.181–1.823	0.0003
Hemoglobin (per one g/dL)	0.863	0.763–0.976	0.0186
Serum albumin (per one g/dL)	0.622	0.405–0.954	0.0284
Total lymphocyte count (per one/μL)	0.9999	0.9995–1.0002	0.4603
eGFR (per one mL/min/1.73 m^2^)	0.996	0.985–1.006	0.4349
CRP (per one mg/dL)	1.069	0.998–1.144	0.0331

BMI: body mass index; eGFR: estimated glomerular filtration rate; CRP: C reactive protein; HR: hazard ratio; CI: confidence interval.

**Table 4:** Receiver operating characteristic curve analysis of independent factors in the multivariate analysis for the grip strength decline.

Variables	AUC	Sensitivity (%)	Specificity (%)	Reference Value
Age (years)	0.74	72.9	65.0	72
BMI (kg/m^2^)	0.60	68.3	51.3	22.6
SARC-F score	0.73	64.3	77.5	1
Hemoglobin (g/dL)	0.70	65.6	66.2	12.4
Serum albumin (g/dL)	0.70	80.8	49.8	4.0
CRP (mg/dL)	0.60	44.0	76.5	0.46

BMI, body mass index; CRP, C reactive protein; AUC, area under the receiver operating characteristic curve.

## Data Availability

The data are not publicly available due to personal information of our study cohort.

## Abbreviations

Ga-D: gastrointestinal disease, QOL; quality of life, GS; grip strength, BMI; body mass index, ROC; receiver operating characteristic curve, IQR; interquartile range, AUC; area under the receiver operating characteristic curve, U-GD; upper gastrointestinal disease, L-GD; lower gastrointestinal disease, BP-D; biliary and pancreatic disease, L-D; liver disease, CRP; C reactive protein.
